# 

**Published:** 1911-06

**Authors:** 


					Uj  JUNE, 1911.
Vol. XXIX. No. 112.
THE
BRISTOL
MEDICO-CHIRURGICAL
TOURNAti
%-/ PUBLISHED UNDER THE AUSPTCES\OF
THE BRISTOL MED1C0-CHIRURGj
I BR ARN
UL a-. 1911
&??r$RAL,si!
: R. SHINGLETON SMITH, M.D., B.Sc.
WITH WHOM ARE ASSOCIATED
J. MICHELL CLARKE, M.A., M.D.,
J. LACY FIRTH, M.S., M.D., JAMES SWAIN. M.S., M.D.
P. WATSON WILLIAMS, M.D., Assistant Editor.
Editorial Secretary: J. A. NIXON, B.A., M.B. v
rJSM&r
BRISTOL: J. W. ARROW SMITH LTD.
London : J. & A, Churchill, 7 Great Marlborough Street.
i
Price One Shilling and Sixpence.

				

